# Mutations in the voltage-gated sodium channel gene of anophelines and their association with resistance to pyrethroids – a review

**DOI:** 10.1186/1756-3305-7-450

**Published:** 2014-10-07

**Authors:** Ana Paula B Silva, Joselita Maria M Santos, Ademir J Martins

**Affiliations:** Laboratório de Malária e Dengue, Instituto Nacional de Pesquisas da Amazônia, Av. André Araújo, 2936, Petrópolis, CEP 69067-375 Manaus, Amazonas Brazil; Laboratório de Fisiologia e Controle de Artrópodes Vetores, Instituto Oswaldo Cruz, FIOCRUZ, Rio de Janeiro, Brazil; Instituto Nacional de Ciência e Tecnologia em Entomologia Molecular, Rio de Janeiro, Brazil

**Keywords:** *Anopheles*, Sodium channel, Malaria, Pyrethroids, Resistance, *kdr*

## Abstract

Constant and extensive use of chemical insecticides has created a selection pressure and favored resistance development in many insect species worldwide. One of the most important pyrethroid resistance mechanisms is classified as target site insensitivity, due to conformational changes in the target site that impair a proper binding of the insecticide molecule. The voltage-gated sodium channel (Na_V_) is the target of pyrethroids and DDT insecticides, used to control insects of medical, agricultural and veterinary importance, such as anophelines. It has been reported that the presence of a few non-silent point mutations in the *Na*_*V*_ gene are associated with pyrethroid resistance, termed as ‘*kdr*’ (*knockdown resistance*) for preventing the knockdown effect of these insecticides. The presence of these mutations, as well as their effects, has been thoroughly studied in *Anopheles* mosquitoes. So far, *kdr* mutations have already been detected in at least 13 species (*Anopheles gambiae*, *Anopheles arabiensis*, *Anopheles sinensis*, *Anopheles stephensi*, *Anopheles subpictus*, *Anopheles sacharovi*, *Anopheles culicifacies*, *Anopheles sundaicus*, *Anopheles aconitus*, *Anopheles vagus*, *Anopheles paraliae*, *Anopheles peditaeniatus* and *Anopheles albimanus*) from populations of African, Asian and, more recently, American continents. Seven mutational variants (L1014F, L1014S, L1014C, L1014W, N1013S, N1575Y and V1010L) were described, with the highest prevalence of L1014F, which occurs at the 1014 site in Na_V_ IIS6 domain. The increase of frequency and distribution of *kdr* mutations clearly shows the importance of this mechanism in the process of pyrethroid resistance. In this sense, several species-specific and highly sensitive methods have been designed in order to genotype individual mosquitoes for *kdr* in large scale, which may serve as important tolls for monitoring the dynamics of pyrethroid resistance in natural populations. We also briefly discuss investigations concerning the course of *Plasmodium* infection in *kdr* individuals. Considering the limitation of insecticides available for employment in public health campaigns and the absence of a vaccine able to brake the life cycle of the malaria parasites, the use of pyrethroids is likely to remain as the main strategy against mosquitoes by either indoor residual spraying (IR) and insecticide treated nets (ITN). Therefore, monitoring insecticide resistance programs is a crucial need in malaria endemic countries.

**Electronic supplementary material:**

The online version of this article (doi:10.1186/1756-3305-7-450) contains supplementary material, which is available to authorized users.

## Introduction

### The global situation of malaria and its vectors

Malaria is one of the most serious and complex health problems faced by humanity. Besides that, it has become a threat for social and economical development in tropical and subtropical regions, specially given the decrease in work capacity of the affected victims [1]. According to the World Health Organization (WHO), approximately 207 million cases of malaria were reported in 2012, with an estimate of 627,000 deaths, with the highest incidence rates observed in Africa (80%), Asia (15%) and the Americas (14%) [2]. Among the factors contributing to this scenario, it is possible to highlight the absence of an effective antimalarial vaccine, the distribution of drug-resistant *Plasmodium*, the development of insecticide resistance in vector mosquitoes, as well as ecological, socio-economic and medical-sanitary factors [3, 4]. Mosquito resistance to at least one insecticide used for malaria control has been identified in 64 countries [5]. Malaria vectors are part of the *Anopheles* genus, including nearly 484 species, distributed in seven subgenera [6], 70 of which showing vectorial competence for human malaria [7], with 41 of them being considered as dominant vector species [8] (Table 1).

**Table 1:** **List of 41 dominant vector species by area**

Continent	Anopheline species
**Africa**	*Anopheles arabiensis*, *Anopheles funestus*, *Anopheles gambiae*, *Anopheles melas*, *Anopheles merus, Anopheles moucheti* e *Anopheles nili*
**Asia**	*Anopheles barbirostris*, *Anopheles lesteri*, *Anopheles sinensis*, *Anopheles aconitus*, *Anopheles annularis*, *Anopheles balabacensis*, *Anopheles culicifacies*, *Anopheles dirus*, *Anopheles farauti*, *Anopheles flavirostris*, *Anopheles fluviatilis*, *Anopheles koliensis*, *Anopheles leucosphyrus*, *Anopheles maculatus*, *Anopheles minimus*, *Anopheles punctulatus*, *Anopheles stephensi*, *Anopheles subpictus* e *Anopheles sundaicus*
**Americas**	*Anopheles freeborni*, *Anopheles pseudopunctipennis*, *Anopheles quadrimaculatus*, *Anopheles albimanus*, *Anopheles albitarsis*, *Anopheles aquasalis*, *Anopheles darlingi*, *Anopheles marajoara* e *Anopheles nuneztovari*

Compiled from Sinka *et al.* [9–11].

### Use of insecticides against malaria vectors

The strategic tools to fight malaria are oriented towards two principal directions: (i) prevention, by means of controlling vector mosquitoes; and (ii) case management, through malaria diagnosis and treatment with effective medicines, being the former considered as the most effective [12]. The techniques for controlling vector mosquitoes are didactically classified as: mechanical (elimination of breeding sites), biological (use of predators or parasitoids) or chemical (application of synthetic insecticides) [13, 14]. The development of chemical insecticides that remain active for long periods of time was one of the most relevant breakthroughs of the 20th century [15] and nowadays they still play an important role in the control of disease vectors and plagues in agriculture.

There are four main groups of neurotoxic insecticides permitted to be used for public health purposes, classified according to their chemical nature and mode of action: organochlorines, organophosphates, carbamates and pyrethroids. The first insecticide used against anophelines was the DDT, an organochlorine firstly used in Naples in 1944 against a typhus epidemic [15]. In 1995, WHO proposed the global eradication of malaria based on the spraying of DDT inside the houses. Highly efficient and inexpensive, it was able to decimate populations of vectors on a global scale. However, the development of environmental and sanitary problems, coupled with the emergence of resistance, resulted in the prohibition of the product in many countries [16]. In spite of that, after the “Stockholm Convention on Persistent Organic Pollutants” in 2007, DDT was reestablished in restricted areas with high malaria transmission, such as in African locations [17].

The organophosphates (malathion, temephos, fenitrothion etc.) were developed in the 1940s and have been used ever since as insecticides, herbicides and plant growth regulators. Despite being biodegradable and non-cumulative, they have disadvantages, like chemical instability and high toxicity for vertebrates [18]. The carbamates, also referred to as methylcarbamates for deriving from the methylcarbamic acid [19], are compounds used as insecticides, nematicides and acaricides. They have low environmental persistence and are less toxic to living organisms than organochlorines. Due to their wide use in agriculture, they were incriminated as food, water and air contaminant agents, with adverse effects in humans and other animals [20]. Around 1970, synthetic pyrethroids were released as a class of insecticides considered more efficient and less toxic. These insecticides raised the attention for presenting higher lethal capacity against insects, requiring only small doses of the product for satisfactory effects [21]. Consequently, pyrethroids virtually substituted/supplemented the use of other classes in many pest control areas, representing nearly 23% of the chemical insecticides market, more than one fourth of the world market [22].

Pyrethroids are synthetic analogues of the chrysanthemic acid (pyrethrins I) and pyrethric acid (pyrethrins II) ester insecticides, naturally found in leaves of *Chrysanthemum cinerafolis*. They are chemically distinguished as type I, compounds that lack an alpha-cyano group, like permethrin, and type 2, with an alpha-cyano group, like deltamethrin [23]. They are biodegradable, non-cumulative insecticides that rarely cause acute intoxication in birds and mammals [24]. Currently, malaria control basically depends upon this insecticide class, which has been widely employed in indoor residual spraying (IRS) and also to control agricultural pests worldwide. Besides, pyrethroid is the only class approved by the World Health Organization Pesticide Scheme (WHOPES) for mosquito net impregnation (Insecticide Treated Net – ITN; Long Lasting Insecticide Treated Net - LLIN) [1, 25, 26].

IRS is a method in which residual insecticides are applied on the surface of walls and ceilings of houses [27]. Based on this strategy it is expected that the mosquitoes, after feeding on blood, rest on these surfaces and remain long enough to absorb a lethal dose of the insecticide. ITN is a mosquito net that repels, incapacitates or kills mosquitoes that come into contact with the insecticide impregnated in the net material, being both a chemical and a physical barrier against insects. There are two ITN categories: conventional nets and LLIN [27, 28].

The initial success of insecticide based strategies caused the optimistic sensation that the elimination of malaria as a public health concern would be possible through the elimination of its vectors. However, these strategies are threatened today, due to the emergence of vector populations resistant to insecticides. Since new classes of alternative, equally interesting insecticides are not yet available on the market, the selection for resistance tends to continue increasing, unless effective management strategies are implemented [29].

## Review

### Mode of action of pyrethroids

Pyrethroids, such as DDT and its analogues, belong to a group of neurotoxins that share a similar mode of action. They all target Na_V_, which is present in cells of the central and peripheral nervous systems (neurons, myocytes, endocrine cells and ovaries), changing the kinetics of propagation of nerve impulses [22]. Structurally, Na_V_ is an integral transmembrane protein, composed of four homologous domains (I-IV), each of them composed of six helices (S1-S6) connected by *loops*. The segments S5, S6 and the S5-S6 *P-loops* form a central aqueous pore, and the S1-S4 helices of each domain unite to form four independent voltage-sensitive domains [30, 31]. The *A. gambiae Na*_*V*_ alpha subunit gene comprises an ORF (Open Reading Frame) with 6,417 nucleotides that encodes 2,139 amino acids, resulting in a protein with a molecular mass of 240 kDa. This gene, located at the *para* (paralysis) loco of the X chromosome, is composed of 35 exons, including two duplicated exons, and 32 introns, which transcribes for different messenger RNAs (mRNA) through alternate *splicing* [31].

The effects of pyrethroids are stereospecific and two different Na_V_ binding sites were identified. The first was proposed by O’Reilly *et al.* [32], in which IIS5 and IIIS6 helices would play an important role in the interaction with the insecticide molecule and the additional link in the IIS4-S5 linker would explain the higher potency of pyrethroids compared with DDT. The second was suggested by Du *et al.* [33], where the binding site would be a type of “pocket” formed by the IS4-S5 linker and the helices IS5 and IIS6. For both models, the selective effect of the insecticide would be explained by the non-conservation of the amino acids of these regions between arthropods and other animals.

### Pyrethroid resistance mechanisms

Insecticide resistance can be defined as the ability of individuals of a species to withstand doses of toxic substances, that would be lethal for most individuals of a population [34]. It is, therefore, a milestone in the change of the genetic composition of a given population, in response to the selection pressure. This is a typical case of Natural Selection, which consists in the increase of the relative frequencies of some “pre-adapted” individuals present in a population, resulting from the constant application of the same chemical product [35]. Intensive and extensive use of chemical insecticides has selected populations resistant to these compounds [36]. The resistance phenomenon has been observed in more than 500 insect species around the world, among which more than 50 are anophelines [37]. According to WHO [5], resistance to at least one insecticide had been identified in 64 malaria-endemic countries. Resistance to pyrethroids seems to be the most widespread. Two main mechanisms are incriminated as responsible for the pyrethroid resistance: metabolic resistance and target-site insensitivity [38, 39].

Metabolic resistance occurs when high activity of one or more enzymes results in a sufficient portion of insecticide being sequestered or detoxified before reaching its target and promoting the desired effect [38]. It occurs due to the increase in the number of available molecules (genetic amplification or hyperactivation of the gene expression) or through mutations in the coding gene portion of the enzyme, producing the more efficient metabolization of the insecticide [37, 40, 41]. This mechanism is highly complex, although recent advances have been characterizing the main enzyme genes responsible for the detoxification, paving the way for the development of molecular markers for the resistance [42]. Three main enzyme superfamilies are involved in the detoxification process: Esterases, Mixed Function Oxidases (MFO, or simply P450) and Glutathione S-Transferases (GST) [37]. Colorimetric biochemical trials are widely employed to detect changes in the activity of detoxification enzymes. In this test, the enzymatic activity of a natural population is compared with the control lineages ones, using specific substrates for each enzymatic family [43].

On a transcriptional level, more recently microarray assays have gained prominence in the investigation of metabolic resistance. In this technique, the detoxification chips (or *detox chips*) compare the expression of virtually all genes of the families related to the metabolism of insecticides (GSTs, MFOs, Esterases), between resistant and susceptible mosquitoes. In addition to these main families, the expression of other genes are evaluated, such as some related to redox metabolism, involved in the protection against free radicals [44]. The analysis of the gene expression through *detox chip* in *A. gambiae* showed high activity of GST genes (*GSTE2*), P450 (*CYP6Z1* and *CYP325*) and peroxidases in DDT resistant mosquitoes [44]. Genes with anti-oxidizing function (Superoxide dismutase, GST, Peroxidase and P450) were differently expressed in deltamethrin-resistant populations of *A. arabiensis* in Cameroon [45]. High expression of *CYP6P3*, a gene of the P450 family, was observed in permethrin-resistant populations of *A. gambiae* [46]. Differential expression was also observed in *A. funestus*, whose P450 genes (*CYP6P9*, *CYP6M7*) and *COI* (from the redox system) were more expressive in resistant individuals [47].

Resistance based on target-site insensitivity occurs when there is an alteration in the molecules that directly interact with the insecticide, making it less toxic or inefficient [42, 43, 48]. Since insecticide targets are structural molecules of the nervous system, highly conserved throughout evolution, few alterations are permissive without the loss of their physiological functions. Thus, it is common that the mutations selected for resistance occur at homologous sites among different insect species [49]. Target-site insensitivity is the most understood mechanism, and in many cases is the characteristic attributed to the higher portion of the genetic variation related to resistance [50]. In this sense, molecular diagnoses for detection target-site mutations are part of the strategies to monitor insecticide resistance in many malaria control programs [51].

### *Kdr* mutations as a resistance mechanism

Many studies showed that resistance to the knockdown effect of several insect species is associated with point mutations in the *Na*_*V*_ gene. By definition, the knockdown effect is the loss of coordination and paralysis caused by the insecticide, which are often accompanied by spasms and tremors [22]. This resistance mechanism was first observed in the housefly *Musca domestica* [52], where later it was suggested that the substitution of one amino acid leucine by phenylalanine in the hydrophobic segment IIS6 (L1014F) resulted in a moderate increase of DDT resistance, termed as the *kdr* mutation (*knockdown resistance*). In *Anopheles* the homologous L1014F *kdr* mutation was first identified in lineages of *A. gambiae* resistant to pyrethroids [53] and since then it has also been detected in a series of other anophelines [54–59]. Still in the 1014 site, another substitution, leucine by serine (L1014S), was identified in *A. gambiae,* also associated with the *kdr* phenotype [60]. The mutations L1014F and L1014S were first observed in populations of West and East Africa, respectively. Therefore, the former is sometimes referred to as *kdr-w* (*kdr-west*), and the latter, as *kdr-e* (*kdr-east*) [61]. In any case, it is noticeable that the distribution of these mutations is strongly related to sibling species of the *Anopheles gambiae* complex [62].

In Asian *A. sinensis* populations, in addition to the L1014F/S substitutions, the mutations L1014C and L1014W were reported, changing the amino acid leucine to cysteine and to tryptophan, respectively. Additionally, in the site immediately before the one of the classical *kdr* mutation, an N1013S substitution occurs, changing the amino acid asparagine to serine [63, 64]. In Indian *A. culicifacies* populations, also in addition to the L1014F/S substitutions, a new mutation in the site 1010 was described, substituting valine by leucine (V1010L) [65].

Another mutation in the Na_V_ of *Musca domestica*, which substitutes methionine by threonine in 918 position, corresponding to the loop between IIS4-S5 segments, synergic to the classical L1014F mutation, was associated with high levels of DDT and pyrethroid resistance, thus being referred to as *super kdr* [66]. An analogous situation was observed in other insect species, such as in the horn fly *Haematobia irritans*[67], green peach aphid *Myzus persicae* [68, 69], onion thrips *Thrips tabaci* [70] and in the moth *Tuta absoluta* [71]. However, there are still no records of homologous substitutions in anophelines.

Based on the current molecular techniques, it was possible to identify and map the distribution of *kdr* mutations among a wide range of *Anopheles* species around the world. Since it was first described in 1998 [53], the identification of changes in the *Na*_*V*_ gene in the *Anopheles* genus has been widely monitored, in a way that we were able to recorded about 98 references published until the end of 2013 (Table 2).

**Table 2:** **Anopheline species with** ***kdr*** **mutations detected**

Species	Locality	Type of mutation	References
*Anopheles gambiae*			
	Ghana	L1014F/N1575Y/L1014S	[62, 72–77]
	Nigeria	L1014F/L1014S	[56, 62, 78–80]
	Burkina Faso	L1014F/N1575Y/L1014S	[53, 57, 62, 72, 77, 81–87]
	Cameroon	L1014F/N1575/L1014S	[54, 62, 77, 88–96]
	Ivory Coast	L1014F/L1014S	[53, 55, 62, 83, 97–99]
	Kenya	L1014S	[60, 100–106]
	Angola	L1014F/L1014S	[62, 107]
	Benin	L1014F/N1575Y/L1014S	[62, 77, 108–113]
	Mali	L1014F/L1014S	[114, 115]
	Chad	L1014F	[116]
	Congo	L1014F/L1014S	[117, 118]
	Equatorial Guinea	L1014F/L1014S	[54, 119]
	Gabon	L1014F/L1014S	[62, 120, 121]
	Senegal	L1014F/L1014S	[62, 122]
	Uganda	L1014F/L1014S	[123–126]
	Tanzania	L1014S	[127]
	Burundi	L1014S	[128]
	Liberia	L1014F	[129]
	Niger	L1014F	[130]
*Anopheles arabiensis*			
	Sudan	L1014F/L1014S	[131–134]
	Burkina Faso	L1014F/L1014S	[57, 82, 84, 86, 135, 136]
	Ethiopia	L1014F	[58, 137]
	Kenya	L1014S	[102, 104]
	Benin	L1014S	[112]
	Tanzania	L1014F	[138]
	Uganda	L1014S	[125]
*Anopheles sinensis*			
	China	L1014F/L1014S/L1014C/L1014W/N1013S	[64, 139–141]
	Korea	L1014F/L1014C	[142]
	Laos	L1014S	[143]
	Cambodia	L1014S	[143]
	Vietnam	L1014S	[143]
*Anopheles stephensi*			
	Dubai	L1014F	[144]
	India	L1014F/L1014S	[145, 146]
*Anopheles subpictus*			
	Sri Lanka	L1014F	[147]
	Indonesia	L1014F	[148]
*Anopheles albimanus*			
	Mexico	L1014F	[149]
	Nicaragua	L1014C	[149]
	Costa Rica	L1014C	[149]
*Anopheles sacharovi*			
	Turkey	L1014F/L1014S	[150]
*Anopheles culicifacies*			
	India	L1014F/L1014S/V1010L	[65, 151]
*Anopheles sundaicus*			
	Indonesia	L1014F	[148]
*Anopheles aconitus*			
	Indonésia	L1014F	[148]
*Anopheles vagus*			
	Indonesia	L1014F	[148]
	Laos	L1014S	[143]
	Cambodia	L1014S	[143]
	Vietnam	L1014S	[143]
*Anopheles paraliae*			
	Laos	L1014S	[143]
	Cambodia	L1014S	[143]
	Vietnam	L1014S	[143]
*Anopheles peditaeniatus*			
	Laos	L1014F/L1014S	[143]
	Cambodia	L1014F/L1014S	[143]
	Vietnam	L1014F/L1014S	[143]

**Figure 1:**
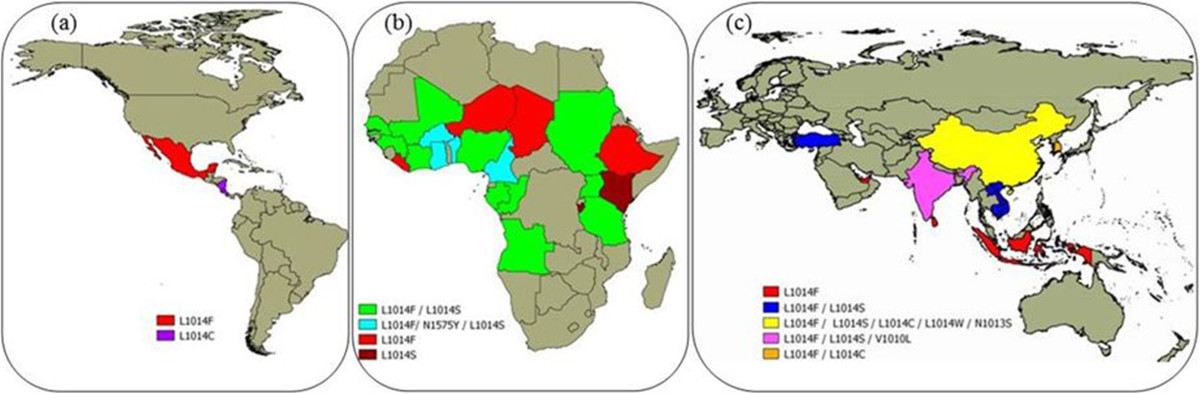
**Distribution of** ***kdr*** **mutations in** ***Anopheles*** **mosquitoes around the world: (a) America, (b) Africa, (c) Asia.**

So far, *Na*_*V*_ mutations were described in at least 13 different anophelines. *A. gambiae* was the most studied (62 records), showing three mutational variants (L1014F, L1014S and N1575Y), detected in 19 out of 54 countries in Africa (Figure 1). Following, the African *A. arabiensis* presented 17 records, showing two variants (L1014F and L1014S) detected in seven countries. *A. sinensis* was the third one, with six records. Surprisingly, it showed the highest number of *kdr* variants (L1014F, L1014S, L1014C, L1014W and N1013S), distributed in five Asian countries, mostly detected in China. According to Kang *et al.* [142], this fact is related to the high population size and to the wide geographical distribution of the species, which tends to increase the genetic variability.

Among other Asian species, *A. stephensi* showed three records of two variants (L1014F and L1014S), detected in Dubai and India. *A.subpictus* (L1014F), *A. culicifacies* (L1014F, L1014S and V1010L) and *A. vagus* (L1014F) showed two records; while *A. sacharovi* (L1014F/L1014S), *A. sundaicus* (L1014F), *A. aconitus* (L1014F), *A. paraliae* (L1014S) and *A. peditaeniatus* (L1014F/L1014S) had just one record. The presence of *kdr* mutations in the Americas was observed only in *A. albimanus*, for the variants L1014F and L1014C in populations from Mexico, Nicaragua and Costa Rica (Table 2).

A survey on the geographical distribution of *kdr* mutations in African populations of *A. gambiae*, conducted by Pinto *et al.* [61], detected the presence of the variant L1014F in west countries (*kdr-w*), from Nigeria to Senegal, the presence of L1014S (*kdr-e*) in the East (Kenya), and both mutations occurring in the Midwest, comprising Angola, Gabon, Equatorial Guinea and Cameroon. This same distribution pattern was reported one year later by Santolamazza *et al.* [62]. The occurrence of both mutations is currently found, sympatrically, in several African countries. Exceptions were Niger, Ethiopia, Chad and Liberia, which reported the presence of L1014F only, and Burundi and Kenya with L1014S only (Figure 1).

It is noteworthy that the *A. gambiae* complex is composed of seven sibling species: *A. gambiae s.s.*, *A. arabiensis*, *A. melas*, *A. merus*, *Anopheles quadriannulatus* species A, *A. quadriannulatus* species B and *Anopheles bwambae*. They are morphologically indistinguishable, however, they can be classified according to fixed and polymorphic chromosomal inversions [152]. The classical molecular forms are *Savannah*, *Mopti*, *Bamako*, *Forest* and *Bissau*, according to paracentric inversions of the second chromosome of *A. gambiae s.s.* [153]. The mutation L1014F was firstly described in the *Savannah* form of *A. gambiae* populations, also known as S form, and until mid-1999 this mutation had not occurred in sympatry with the *Mopti* form (M form) [154]. However, later studies identified its presence also in the M form, possibly resulting from genetic introgression from the S form [81, 155]. Introgression was also suggested by Tripet *et al.* [114] when the *kdr* allele was detected in the *Bamako* form. On the other hand, a new independent mutational event could explain the emergence of the *kdr* mutation in *A. arabiensis* [135].

Despite 15 years of research, some doubts still arise with respect to the *kdr* mutations, especially if they are indeed correlated with the resistant phenotype. One of the techniques adopted to test this association is the employment of bioassays with insecticides (WHO cones, bottle test, ITN, LLIN) followed by the genotyping of *kdr* mutation between dead and surviving mosquitoes after the test. In other words, it is aimed to test whether the mutation frequency is higher among resistant than the susceptible individuals. In our survey, out of the 98 studies here considered, 63 (64.3%), conducted bioassay followed by genotyping, correlating the mutation with insecticide resistance. Among them six detected the involvement of more than one mechanism of resistance (target site and metabolic alterations) [78, 82, 88, 100, 139, 140] and two only suggested their occurrence [123, 129]. On the other hand, six studies (9.5%) did not associate the occurrence of Na_V_ mutations with knockdown resistance [58, 107, 119, 131, 136, 143]. In these cases, the lack of a “mutation *versus* resistance” association was suggested due to low sample size [107], mutation similarly distributed between dead and surviving individuals in the insecticide bioassay [58, 131, 136, 143] or mutation among susceptible individuals [119]. Nevertheless, in this last example the authors recognized that the bioassays were performed outside the WHO recommended standards. Lastly, in 28 studies (28.6%) only the genotyping of field samples was performed, considering the presence of the mutation as enough evidence for resistance.

### Association between ITN and *kdr* mutation

The use of ITNs/LLINs treated with pyrethroids is an important tool to reduce morbidity and mortality caused by malaria [26]. According to a survey performed by Lengeler *et al.* [156], the implementation of this strategy in Sub-Saharan Africa, between 1986 and 2003, was able to reduce morbidity by 50% and the infant mortality by 17%. In Kenya, for instance, the employment of ITNs was able to prevent infant mortality in an area with high malaria transmission [25]. However, the maintenance of this efficiency is still a controversial issue nowadays, given the occurrence of highly resistant anopheline populations. There are several records indicating good results of pyrethroid treated materials where *kdr* mutation had been identified, such as in Nigeria [157], where the LLINs were efficient at killing or reducing the blood feeding of *A. gambiae*, Mali [115], Benin [108, 158] and Uganda [124]. On the other hand, a reduction in the susceptibility of *A. gambiae* populations subjected to ITNs was observed in Uganda [159]. Besides that, increases in *kdr* frequency were evidenced for this same species after the distribution of LLINs in Kenya [101], Niger [130], Senegal [122] and Benin [160].

The most recent update of WHOPES continues indicating only pyrethroids (deltamethrin, alphacypermethrin, permethrin and a combination of deltamethrin or permethrin and piperonyl butoxide – PBO) for LLINs [161]. However, given the possibility of loss of effectiveness caused by resistance, the development of mosquito nets impregnated with other classes of insecticides is a promising alternative. A study conducted with mosquito nets impregnated with chlorpyrifos-methyl (organophosphate) and lambdacyalothrin (pyrethroid), showed that, alone or combined, they were efficient at killing or reducing blood feeding of *A. gambiae* from the Ivory Coast, even in areas with high *kdr* and *ace-I*^*R*^ mutation frequencies. This *ace-1*^*R*^ mutant allele belongs to the acetylcholinesterase gene, conferring resistance to organophosphates [162].

#### Association between *Plasmodium* infection and insecticide resistance

Regardless of the extensive literature concerning *kdr* mutations and their association with resistance to insecticides, few reports have presented their impact on malaria transmission dynamics, i.e., on the ability of mosquitoes to transmit malaria. Infection rate and oocyst burden are two of the five factors that determine the vectorial capacity of mosquitoes [163]. The response to *Plasmodium* exposure in vectors is modulated by the mosquito’s innate immune system. In *A. gambiae,* for example, changes in its global gene expression patterns are expressed upon *Plasmodium* infection [164]. Exposure to pyrethroids, in turn, induces metabolic changes that alters the immune response [165] and may therefore affect the outcome of *Plasmodium* infection.

An insecticide susceptible strain of *A. funestus* showed greater ability to become infected with *Plasmodium berghei* than its resistant counterpart [166]. In *A. gambiae*, infection with this same parasite increased the expression level of *CYP6M2,* a gene related with metabolic resistance [164]. In relation to the possible impacts of *kdr* mutation on vector competence, few records are available and are sometimes conflicting. For instance, neither positive or negative correlation was found between the occurrence of *kdr* and *ace-1*^*R*^ alleles with infection of *Plasmodium falciparum* in *A. gambiae* natural populations from Benin [109].

Other studies, however, showed that the presence of both resistant alleles could be associated with increased prevalence of *Plasmodium* infection in an *A. gambiae* resistant strain. Additionally, individuals carrying the *kdr* mutation had increased prevalence of sporozoites, which is likely to impact on parasite transmission [167]. Given the dissemination of *kdr* mutation in natural populations, similar studies should be conducted in order to better understand the impact of insecticide resistance on vector competence.

#### Molecular tools for *KDR* mutation diagnosis

The resistance phenomenon can be studied on many levels, from biological assays in order to evaluate the susceptibility/resistance status to biochemical and molecular characterizations able to infer the mechanisms and effective genes selected for resistance. Currently, the development of tools for genetic screening of natural populations on a large scale, are aimed to predict the predisposition of those populations to develop insecticide resistance.

Thus, the identification of genetic markers associated with resistance were included in the priorities of the WHO Global Plan for Insecticide Resistance Management (GPIRM) [5]. In this sense, the identification of *kdr* genetic markers truly associated with pyrethroid resistance, as well as the improvement of existent diagnostic assays are constantly in the course of studies in this field. DNA based genotyping techniques have as main advantages the high sensitivity and the capacity to distinguish between homo and heterozygous individuals [37]. The principal methods employed in the detection of *kdr* mutations are listed in Table 3, with emphasis on the equipment required for each technique.

**Table 3:** **Molecular methods used for detecting kdr mutations**

Method	Equipment required	Mutation	References
Allele-Specific Polymerase Chain Reaction (AS-PCR)	PCR thermocycler, electrophoresis and imaging equipments	L1014F/S/C	[53, 60]
Heated Oligonucleotide Ligation Assay (HOLA)	PCR thermocycler, ELISA plate reader	L1014F/S	[168]
Sequence-Specific Oligonucleotide Probe – Enzyme-Linked ImmunoSorbent Assay (SSOP-ELISA)	PCR thermocycler, shaking incubator and ELISA plate reader	L1014F/S	[138]
PCR Sequence Specific Oligonucleotide Probe Assay (PCR-Dot Blot)	PCR thermocycler, shaking incubator and nylon membrane	L1014F/S	[169]
Fluorescence Resonance Energy Transfer (FRET)/Melt Curve Analysis (MCA)	Real-Time PCR thermocycler	L1014F/S	[125]
PCR Elongation with Fluorescence	PCR thermocycler and electrophoresis equipments	L1014F/S	[170]
High Resolution Melt (HRM)	Real-Time PCR thermocycler	L1014F/S	[171]
Allele-Specific Loop-Mediated Isothermal Amplification (AS-LAMP)	Turbidimeter and water bath	L1014F	[172]
Polymerase Chain Reaction-Restriction Fragment Length Polymorphism assay (PCR-RFLP)	PCR thermocycler	L1014F/C	[141]
Primer Introduced Restriction Analysis-PCR assay (PIRA-PCR)	PCR thermocycler, electrophoresis and imaging equipments	L1014F/S	[173]
Multiplex Primer Introduced Restriction Analysis-PCR assay (mPIRA-PCR)	PCR thermocycler and electrophoresis equipments	L1014F/S	[174]
Amplification Refractory Mutation System (ARMS)	PCR thermocycler, electrophoresis and imaging equipments	L1014F	[151]

### Strategies for managing resistance

The evolution of insecticide resistance has become a great threat to chemical products-based malaria control programs due to the strong selection pressure placed on resistance genes [5]. Therefore, strategies for managing resistance to minimize operational obstacles to the use of a given product have gained prominence on the world stage. The resistance management strategies are divided into three groups: management by moderation, management by saturation and management by multiple attack [175].

Management by moderation aims to reduce the selection pressure to conserve susceptible individuals of a given population, by the use of lower dosages of insecticides, higher treatment thresholds, chemicals with shorter residual activity and maintaining unsprayed areas as refuges for susceptible individuals [176]. Even though, peculiarities have to be considered. For instance, a study evaluating the effects of sublethal doses of permethrin in an *A. stephensi* strain showed that lower concentrations were more efficient in increasing the mortality rates [177]. Concerning refuges, it is important to maintain susceptible alleles in a population, mainly in the case of resistant alleles, which carry a fitness cost. However, resistant alleles can also invade untreated areas. This was the case observed in a survey conducted in populations of *A. gambiae* from Burundi, where high frequencies of *kdr* allele were detected in unsprayed areas, due to migration [128].

Management by saturation involves methods that overcome resistance mechanisms present in the insect, by the use of high rates of insecticides, that should kill even resistant individuals, or by the use of chemical synergists [21]. For example, the evaluation of the dosage-dependent effect of permethrin-treated nets in experimental hut trials from Benin showed that nets treated with higher permethrin concentrations provided better blood feeding prevention against pyrethroid-resistant *A. gambiae* [158]. Similar efficiency against pyrethroid-resistant *A. gambiae* populations were observed in a net impregnated with deltamethrin-pyperonil butoxide combination [157, 178].

Finally, the management by multiple attacks involves either mixtures or rotations of insecticides to avoid resistance. This method is based on the concept that insects resistant to one insecticide will be killed by the other component of the mixture and that few insects will be resistant to the entire mixture [176]. A combination of IRS with chlorfenapyr and LLIN impregnated with deltamethrin, in an experimental hut trial from Benin, was effective to provide additional level of transmission control and personal protection against pyrethroid-resistant *A. gambiae* [108]. Similar results were obtained by the use of mosquito nets impregnated with chlorpyrifos-methyl and lambdacyalothrin against *A. gambiae* from Ivory Coast [162].

## Conclusions

After 15 years of intense research, *kdr* mutations were recorded in 13 anopheline species, in natural populations from three continents, revealing the preponderance of this phenomenon in the process of resistance to pyrethroid insecticides, either alone or combined with other mechanisms (e.g., metabolic resistance). These alterations emerged in different species as well as within populations of the same species, and are spreading quickly, given the strong selection pressure exerted by the pyrethroids. Although compounds with new modes of action, such as neonicotinoids and pyrroles, have been introduced in public health, they are still not indicated for IRS and ITN, for instance. The availability of a new generation of environmentally friendly compounds may take as long as the implementation of advanced strategies, likewise, the use of genetically modified mosquitoes. Therefore, the use of pyrethroids has to be severely monitored in order to try to maximize their effectiveness.

## Authors’ original submitted files for images

Below are the links to the authors’ original submitted files for images. Authors’ original file for figure 1

## Abbreviations

Na_V_: Voltage-gated sodium channel
DDT: Dichlorodiphenyltrichloroethane
*Kdr*: Knockdown resistance
WHO: World Health Organization
IRS: Indoor residual spraying
WHOPES: World Health Organization pesticide scheme
ITN: Insecticide treated net
LLIN: Long lasting insecticide treated net
MFO: Mixed function oxidases
GST: Glutathione S-Transferases
PBO: Piperonil-butoxide.
