# Supplementary figures and images for: Mutations in the voltage-gated sodium channel gene of anophelines and their association with resistance to pyrethroids – a review
Source: Parasit Vectors. 2014 Oct 7;7:450. doi: 10.1186/1756-3305-7-450 (PMC4283120; doi:10.1186/1756-3305-7-450)

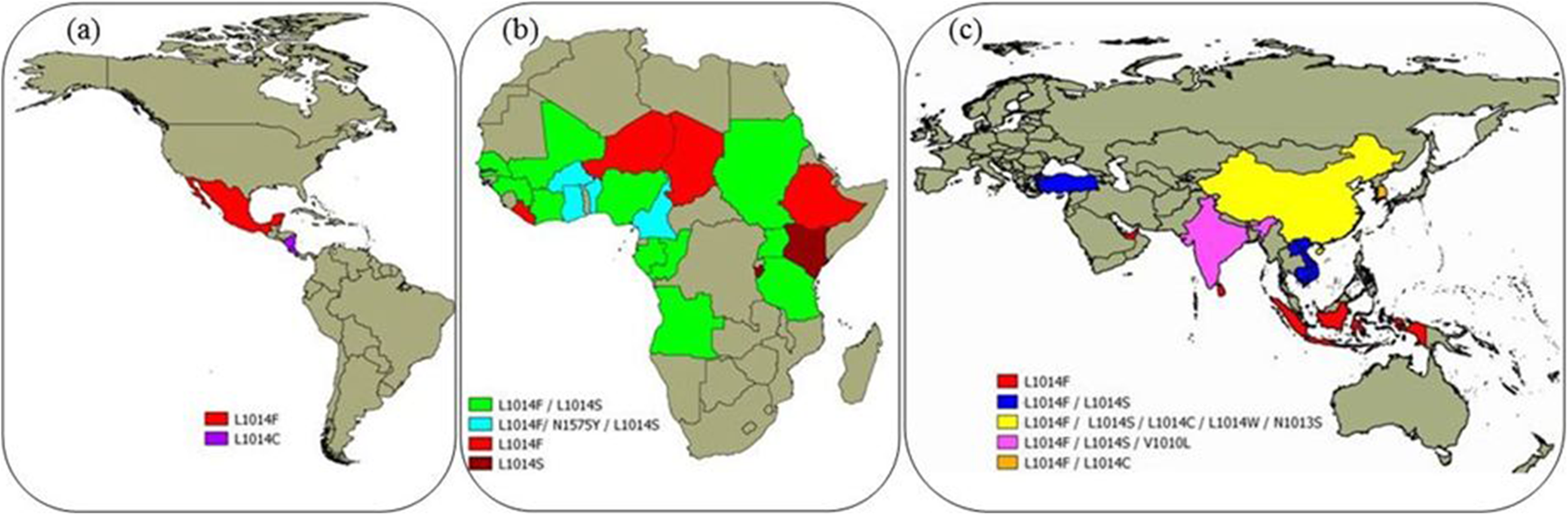

Supplement: Supplementary file 1 — Authors’ original file for figure 1 [file 13071_2014_1625_MOESM1_ESM.tif]
